# Dietary habits and metabolic risk factors for non-communicable diseases in a university undergraduate population

**DOI:** 10.1186/s41043-018-0152-2

**Published:** 2018-08-16

**Authors:** F. A. Olatona, O. O. Onabanjo, R. N. Ugbaja, K. E. Nnoaham, D. A. Adelekan

**Affiliations:** 1 Department of Community Health and Primary Care, College of Medicine, University of Lagos, Lagos, Lagos State Nigeria; 2 Department of Nutrition and Dietetics, College of Food Science and Human Ecology, Federal University of Agriculture, Abeokuta, Nigeria; 3 Department of Biochemistry, Federal University of Agriculture, Abeokuta, Nigeria; 4 Faculty of Health and Human Sciences, Plymouth University, Plymouth, England

**Keywords:** Dietary pattern, Metabolic risk factors, Non-communicable diseases, University undergraduate students, Nigeria

## Abstract

**Background:**

Unhealthy dietary patterns are associated with metabolic changes and increased risk of non-communicable diseases (NCDs), but these associations have not been investigated in representative populations of university undergraduates in low-to-middle income countries (LMICs).

**Methods:**

This study was conducted in the three universities in Lagos State, Nigeria to assess the dietary pattern and metabolic risk factors of NCDs among university undergraduate population. Multistage sampling technique was used to select 506 undergraduates from the universities. Pre-tested questionnaire was used to obtain data on socio-demographic characteristics and dietary patterns. Body mass index and metabolic risk factors (abdominal obesity, dyslipidemias, high blood pressure and hyperglycemia) were assessed following standard procedures. SPSS (version 20) was used for data entry and analysis. Association between variables was determined using chi-square and Fisher’s exact tests.

**Results:**

The mean age was 20.3 ± 3.5 years; 54.7% of them were female. More than one third (37.6%) had no consistent source of income or received less than N10, 000 ($31.7) per month. Less than one third (31.0%) ate three daily meals, 23.0% ate breakfast regularly, and only 2% consumed the recommended daily amount of fruits and vegetables. Almost half (44.0%) ate pastry snacks daily. Refined rice was the commonest cereal (28.2%) consumed while meat was more commonly consumed daily (32.0%) than milk (14.0%) and fish (10.0%). Twenty-nine (29.0%) and 6.2% of the population daily consumed carbonated soft drinks and alcohol, respectively. Prevalence of abdominal obesity (based on waist circumference) was 5% (1.3% in males and 8.4% in females), dyslipidemias (57.3%), pre-hypertension (8.2%), hypertension (2.8%), and pre-diabetes (1.0%). Obesity was positively associated with consumption of alcohol (χ^2^ = 13.299**,** *p* < 0.001).

**Conclusion:**

Unhealthy diets and metabolic risk factors of non-communicable diseases are prevalent in the undergraduate population studied. Well-recognized recommendations regarding adequate consumption of fruits, vegetables, fish, and whole grains should be emphasized in a targeted manner in this population. Carbonated soft drinks and alcohol consumption should be discouraged to stem a rising tide of metabolic risk factors for non-communicable diseases among undergraduate students.

## Background

Unhealthy diets are a key modifiable behavioral risk factor for non-communicable diseases (NCDs). They contribute to the occurrence of a cluster of disorders known as the metabolic syndrome—abdominal obesity, hypertension, dyslipidemia, and disturbed metabolism of glucose or insulin—which in turn accounts for a significant share of the global burden of disease [1]. The presence of the metabolic syndrome increases the risk of developing NCDs such as cardiovascular diseases, diabetes, chronic respiratory diseases, and cancer [2, 3].

In recent decades, the global pattern of unhealthy diets driving the occurrence of metabolic disorders and NCDs has become more important in low-to-middle income countries (LMICs) because of the double burden of diseases in such countries. In high-income countries, an epidemiological transition has effectively occurred but in LMICs, infectious and NCDs now jointly constitute major causes of morbidity and mortality [3]. In South Western Nigeria, for example, previous research revealed that only 60% of university undergraduates consumed the recommended minimum number of servings of grain (cereal) foods while 60%, 85%, and 40% of students did not meet the recommended dietary allowance (RDA) for protein, calcium, and iron respectively. Consumption of meat, milk, and fruits and vegetable was low, and in the same study, body mass index (BMI) classification indicated that 29% of the students were overweight, 6% were obese, and 13% of the male students were underweight [4].

The dietary habits of populations (including young adults) in low-to-middle income countries similarly have rapidly shifted to less-healthy diets (consisting of processed foods, away-from-home food intake, and increased use of edible oils and sugar-sweetened beverages) in line with the global nutrition transition [5]. Young adults are thought to be prone to obesity in the transition from childhood/adolescence to adulthood [5]. The transition to higher education involves a significant life change, including unfavorable changes in health-related behaviors and weight gain for many students [6, 7]. Studies among university students in developing countries have previously shown high prevalence of obesity [8]. Moreover, research has shown that university is a critical period for weight gain in young adults; and that during the transition from secondary school to university, failure of students to adapt to a new environment could have negative consequences on their health behaviors and subsequent weight status [5].

Given the above, it is fair to suggest that adequately sampled university undergraduate student populations offer a useful set of lenses through which population health phenomena and trends in the wider population can be viewed, especially in low-to-middle income countries with typically young adult populations. Although dietary patterns and nutritional status of undergraduates in some Nigerian Universities have been studied [4, 9, 10], the student populations were not sufficiently representative of the Nigerian young adult population neither were the particular influences on university students’ eating habits—such as university characteristics, individual and social environmental determinants [11]—consistent with the predominant patterns in the country’s most populous and diverse populations. Furthermore, much remains to be known of the relationships between the well-studied dietary patterns and profile of metabolic disorders in these samples of LMIC young adult populations [11]. This study therefore assessed the dietary pattern and metabolic risk factors of NCDs among university undergraduate students in all the three universities in Lagos State.

## Methods

The study was cross-sectional in design. The study population consisted of full time undergraduate students in the three universities in Lagos State: University of Lagos (UNILAG), Lagos State University (LASU) and Caleb University. The minimum sample size calculated using Cochran’s formula and prevalence of those who had normal body mass index from a previous study (52%) [4] as ‘p’, the estimated prevalence of expected outcome was 384. The size was however increased to 506 to take care of non-response. A multistage sampling technique was adopted to select the students from the universities. All the three universities were included. Simple random sampling was used to select four faculties from the 12 in UNILAG and LASU and 1 college from Caleb. Using simple random sampling technique (balloting), at least one quarter of the number of departments was selected from each faculty making 11 from UNILAG, 5 from LASU, and 1 from Caleb. Using sampling proportional to size, students were selected across faculties, departments, and levels depending on the total population of students in each faculty, department, and level. Systematic random sampling technique was employed in each class to select respondents. The number required from each class was used to divide the class list to determine the sampling interval. Twenty percent of those who participated in the questionnaire survey were selected using systematic random sampling to determine participants for blood glucose and lipid profiles.

### Data collection

Validated structured interviewer-administered questionnaire was used to obtain data on socio-demographic and economic status, food frequency questionnaire was used to determine food consumption patterns, anthropometric measurements (weight, height, waist circumference, and hip circumference) were made using standard procedures and electronic blood pressure monitor (Omron M2 and M7) were used to measure systolic and diastolic blood pressure. The plasma separated from fasting venous blood samples was used to determine blood glucose and lipid profile spectro-photometrically using Cypress diagnostics kits.

#### Data analysis

IBM SPSS Statistics (originally named Statistical Package for Social Sciences) was used for data entry, validation, and analysis. Relevant summary statistics was generated for the variables. Dietary habits were analyzed using frequency tables and anthropometric indices, blood pressure, lipid profile, and fasting blood glucose were compared with WHO standards and classified [12]. Chi-square test was used to determine associations between categorical variables. Statistical tests were regarded as statistically significant if *p* value < 0.05.

## Results

### Socio-demographic and economic status

Five hundred and ten undergraduates were invited, but only 503 participated in the study giving a response rate of 98.6%. The age of the students ranged from 15 to 41 years, but the modal age group was 19–24 years (57.9%) while the average age of the sample population in years was 20.3 ± 3.5 with 54.7% being females. Most of their parents had completed secondary school education (75%) and lived in self-contained housing units within story buildings (41.6%), but many students (37.6%) received less than 30 USD per month or had no consistent source of income (the equivalent of 30 USD is less than the minimum wage for those in the lowest socioeconomic class in the country).

### Dietary habits and barriers to eating healthy

Only 31.0% of the undergraduates ate three meals in a day. Less than one quarter (23.0%) consumed breakfast regularly, 29.0% drank carbonated soft drinks while almost half of them (46.1%) ate pastry snacks daily. Almost one quarter (22.7%) ate fast foods daily indicating at least 7 meals away from home each week (Table 1).

**Fig. 1:**
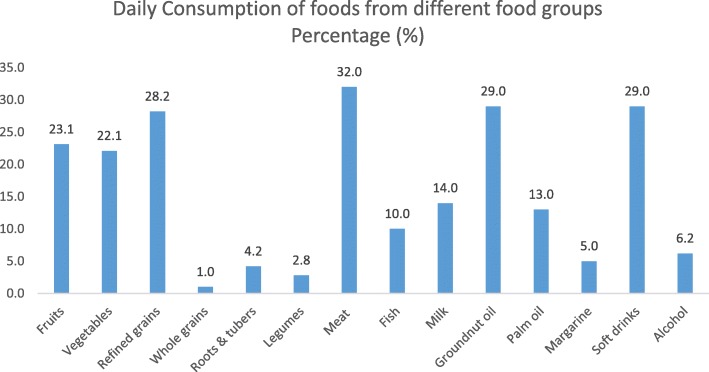
Daily consumption of different foods

**Table 1:** Distribution of respondents according to their dietary habits (*N* = 503)

Dietary habits	Freq	Percentage
No of meals taken daily		
One	35	7.0
Two (2)	287	57.1
Three (3)	156	31.0
Four or more	25	5.0
Daily snack (pastry) intake	221	44.0
Eating in fast food restaurants		
Daily	114	22.7
4–6 times/week	95	18.9
</= 3 times times/week	293	58.4
Daily soft drinks intake	144	29.0
Amount of soft drinks taken daily	(*N* = 144)	
35 ml	99	68.8
=/> 70 ml	45	31.3
Daily fruits and vegetable intake	115	22.9
Portions of fruits and vegetables taken (*N* = 115)		
Less than five	105	91.3
Five or more portions	10	8.7
Daily breakfast consumption	113	22.5

**Fig. 2:**
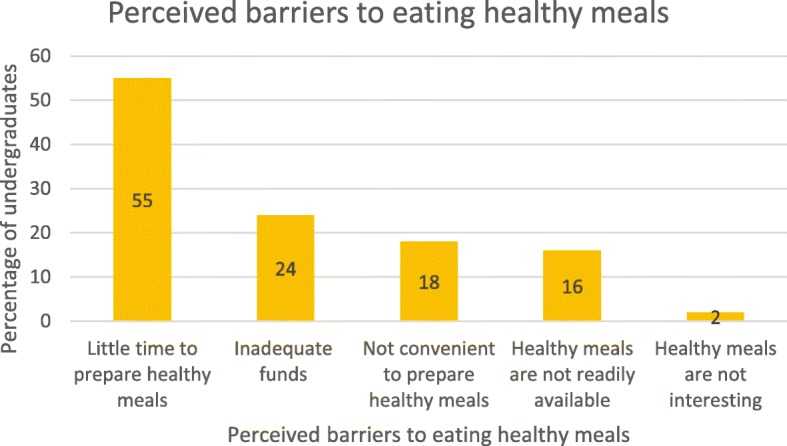
Respondents’ perceived barriers to eating healthy meals

Refined rice and other processed cereals (most likely sugar coated) were the commonest cereals consumed daily (28.2%); whole grains, roots, and tubers were rarely eaten. Meat was more commonly consumed (32.0%) than fish (10.0%); only 30.0% of the study population ate fish at least 3 times per week. Only 22.0% of the respondents ate fruits and vegetables while 6.0% drank alcohol daily. Little time to prepare meals and inadequate funds were cited as the commonest perceived barriers to healthy eating (Figs. 1 and 2).

### Metabolic risk factors

#### Abdominal obesity

The prevalence of abdominal (central) obesity was 5.0% (1.3% in males and 8.4% in females) based on waist circumference and 20% (12.3%) in males and 26.5% in females) based on the waist-to-hip ratio (WHR). Cut-off points were determined using WHO cut-off values for abdominal obesity [13]. Prevalence of abdominal obesity was significantly higher among females compared to males (*p* < 0.001) for both WC and WHR **(**Table 2**).**

**Table 2:** Prevalence of general (BMI) and abdominal (WC and WHR) obesity according to sex

Measure of obesity	Male (*N* = 228)			Female (*N* = 275)			Total			χ^2*^	*p*
	Freq of normal (%)	Freq of overweight (%)	Freq of obesity (%)	Freq of normal (%)	Freq of overweight (%)	Freq of obesity (%)	Freq of normal (%)	Freq of overweight (%)	Freq of obesity (%)		
Body mass index	168(73.7)	36 (16.7)	6 (3.0)	202 (74.0)	44 (16.1)	7 (3.3)	370 (73.9)	82(16.4)	16 (3.2)	0.008	0.996
Waist circumference (WC)	215(94.3)	10 (4.4)	3(1.3)	188(68.4)	64(23.3)	23(8.4)	215(94.3)	74(15.0)	26(5.0)	49.46	< 0.001
Waist-to-hip ratio (WHR)	200(87.7)		28(12.3)	187(68.0)		73 (26.5)	200(87.7)		100 (20.0)	15.12	< 0.001

Body mass index: overweight, > 25–29.99 kg/m^2^; obesity, > 30 kg/m^2^

Waist circumference (WC: overweight: > 94 cm(M); > 80 cm(F), obesity: > 102 cm(M); > 88 cm(F)

Waist-to-hip ratio (WHR): obesity ≥ 0.90 cm(M); ≥ 0.85 cm(F)

*Chi-square test is for a difference in frequency of obesity between males and females as measured by each method

#### Dyslipidemias, high blood pressure, and hyperglycemia

More than half of the students had lower-than-normal levels of HDL-C (57.3%) while 23.8% had levels of LDL-C higher than the upper limit of normal. Only 8.2% and 3.0% had pre-hypertensive systolic (i.e., 120–139 mmHg) and diastolic (80-89 mmHg) blood pressure respectively. The prevalence of hypertension (≥ 140/90 mmHg) was 2.8%. The prevalence of impaired fasting glucose (pre-diabetes) was 1.0%; nobody had diabetes (Table 3).

**Table 3:** Physiological and biochemical measurements of the respondents

Variable	Upper limit of standard values (mmol/liter)	Freq of normal	Percentage	Frequency of disorder	Percentage
Total cholesterol	< 5.2	71	67.6	34	32.4
Triglyceride	< 2	105	100	0	0
HDL-cholesterol	> 1.2	44	42.7	59	57.3
LDL-cholesterol	< 4	80	76.2	25	23.8
Fasting blood glucose	<= 6.1	104	99	1	1
Blood pressure					
	Standard value (mmHg)	Frequency	Percentage		
Systolic					
Optimal BP	< 90	372	74.0		
Normal BP	90–120	76	15.0		
High normal BP	120–139	41	8.2		
Hypertensive	≥140	14	2.8		
Diastolic					
Optimal BP	< 60	432	85.9		
Normal BP	61–80	42	8.3		
High normal BP	81–89	15	3		
Hypertensive	≥ 90	14	2.8		

*HDL* high-density lipoprotein, *LDL* low-density lipoprotein, *BP* blood pressure

#### Factors associated with metabolic risk factors

Consumption of alcohol was the only factor associated with obesity (*p* < 0.001). Family history of obesity, breakfast consumption, pastry snacks, or soft drink intake were not associated with obesity in this study. Raised LDL-C was statistically significantly associated with lower mother’s level of education (*p* = 0.008) and less healthy methods of obtaining water (*p* = 0.018) which are proxy indicators of socio-economic status of parents (Tables 4 and 5).

**Table 4:** Socioeconomic and dietary factors associated with general and abdominal obesity

Factors	General obesity (based on BMI)				Abdominal obesity (based on WC)				Abdominal obesity (based on WHR)			
	Normal BMI *n* (%)	Overweight/obesity *n* (%)	χ^2^	*p* value	Normal WC *n* (%)	Overweight/obesity *n* (%)	χ^2^	*p* value	Normal WHR *n* (%)	Overweight/obesity *n* (%)	χ^2^	*p* value
Income												
Not consistent	87 (85)	15 (15)			87 (84.5)	16 (15.5)			87 (84.5)	16 (15.5)		
N1000–N5000 ($3–15)	41 (83.7)	5 (10.2)			42 (85.7)	7 (14.3)			34 (69.4)	15 (30.6)		
N5001–N10000 ($15–31)	61 (70.9)	17 (19.8)	2.157	0.11	71 (82.6)	15 (17.5)	8.996	0.53	72 (83.7)	14 (16.3)	7.975	0.16
N10001–N20000 (32–61)	128 (76.6)	32 (19.2)			131 (78)	37 (22.0)			136 (81.0)	32 (19.0)		
Above N20000 (> $61)	63 (64.9)	29 (29.9)			72 (74.2)	25 (25.8)			74 (76.3)	23 (23.7)		
Family history of obesity												
Yes	0 (0)	7 (35.0)			13 (65.0)	7 (35.0)			14 (70.0)	6 (30.0)		
No	33 (6.9)	91 (18.9)	4.132	0.127	390 (80.7)	93 (19.3)	3.904	0.15	389 (80.5)	94 (19.5)	1.339	0.25
Pastry snacks intake												
Yes	17 (7.4)	49 (21.2)			187 (80.6)	45 (19.4)			189 (81.5)	43 (18.5)		
No	16 (5.9)	49 (18.1)	1.327	0.515	216 (79.7)	55 (20.3)	1.105	0.58	214 (79.0)	57 (21.0)	0.49	0.48
Soft drinks												
Yes	5 (3.4)	24 (16.6)			50 (59.5)	34 (40.5)			53 (67.1)	47 (26.0)		
No	28 (7.9)	74 (20.8)	5.041	0.08	138 (72.3)	53 (27.7)	5.497	0.06	134 (74.0)	26 (32.9)	1.313	0.25
Breakfast consumption												
Everyday	7 (6.2)	25 (22.1)			85 (75.2)	28 (24.8)			90 (79.6)	23 (20.4)		
Occasionally	17 (6.6)	47 (18.1)	0.885	0.927	218 (83.8)	42 (16.2)	5.701	0.22	211 (81.2)	49 (18.8)	0.415	0.81
Always skipped	9 (7.0)	26 (20.2)			100 (76.9)	30 (23.1)			102 (78.5)	28 (21.5)		
Alcohol consumption												
Yes	4 (4.9)	28 (34.1)			30 (62.5)	18 (37.5)			31 (72.1)	12 (27.9)		
No	29 (6.9)	70 (16.7)	13.299	0.001	158 (69.6)	69 (30.4)	1.557	0.46	156 (71.9)	61 (28.1)	0.001	0.98

**Table 5:** Association between selected socio-economic characteristics and raised LDL-cholesterol

	Normal LDL-C	Raised	Total	χ^2^	*p* value
	*n* (%)	LDL-C *n* (%)	*n* (%)		
Sex					
Male	13 (27.1)	35 (72.9)	48 (100)		
Female	12 (21.1)	45 (78.9)	57 (100)	0.521	0.31
Mother’s level of education					
No education	1 (100)	0 (0)	1 (100)		
Informal education	6 (80.0)	1 (20.0)	7 (100)		
Primary education	3 (33.0)	6 (67.0)	9 (100)		
Secondary education	14 (78.0)	4 (22.0)	18 (100)	21.74	0.008*
Some tertiary	56 (80.0)	14 (20.0)	70 (100)		
Parents’ methods of obtaining water					
Pipe-borne	77 (79.4)	20 (20.6)			
Well	3 (42.9)	4 (57.1)	7 (100)	8.03	0.018*
Stream	0 (0.0)	1 (100)	1 (100)		
Pocket money/income					
Not consistent	13 (81.3)	3 (18.8)	16 (100)		
N1000–N5000 ($3–15)	7 (58.3)	5 (41.7)	12 (100)		
N5001–N10000 ($15–31)	17 (77.3)	5 (22.7)	22 (100)	6.74	0.244
N10001–N20000 (32–61)	32 (76.2)	10 (23.8)	42 (100)		
Above N20000 (> $61)	11 (84.6)	2 (15.4)	13 (100)		

*Fishers’ exact test

Abdominal obesity was statistically significantly associated with hypertension in this study even though high BMI was not associated with it. (*p* < 0001).

## Discussion

Despite the relatively high level of education or income of parents, many students in the study population lived below the poverty line ($1.90) per day—receiving less than N10,000 ($31.76) monthly pocket money or had no consistent source of income—and probably had limited access to healthy diet. Healthier diets cost $1.48/day and $1.54/2000 kcal more than least healthy diet though the cost of disease is much higher [14].

Only 31% ate three meals in a day, while others ate less. This finding is consistent with those of other studies among university students in South Western Nigeria, Korea, and Zimbabwe [4, 15, 16] indicating that most students did not eat the commonly recommended three main meals in a day, and this could lead to malnutrition. Reasons given for not eating healthful meals include little time to prepare such meals and inadequate funds to purchase food from vendors.

Breakfast consumption was poor among the undergraduates; only 23.0% of them consumed breakfast daily. This is similar to the proportion of students who consume breakfast in Hail University in Saudi Arabia (28.0%). Meanwhile, breakfast has been identified as the most important meal in the day necessary for mental work. Whole grains were rarely eaten and refined rice was the commonest cereal consumed daily (28.2%), another finding consistent with the report from the United States of America where college students failed to satisfy whole-grain recommendations [17]. This pattern fits into the global trend of increasing numbers of people consuming refined grains rather than whole grains—the so-called nutrition transition.

Less than one quarter of the respondents ate fruits and vegetables daily, and only a few took at least five recommended portions for adults [2]. Majority of the students consumed insufficient quantities of fruits and vegetables. The consumption pattern of fruit and vegetable was poorer than obtained in other universities in South Western Nigeria where 40% of females and 20% of males ate adequate amount of fruits and vegetables, and another study in Iran where 51.9% of the participants ate fruit every day while only 10% ate it once or twice a week. Another western study revealed that 33.3% of the students met the healthy eating criteria for vegetables and reported that they had eaten: at least three servings of vegetables on 2.7 days and at least two servings of fruit on 3.4 days [4, 18, 19]. Another study among undergraduates in Enugu however corroborated the findings in this study wherein 28.2% and 26.7% consumed fruits and vegetables daily respectively. Kana Sop et al. also found out that undergraduates in Cameroun consumed very little fruits, vegetables, and animal products [19].

Fruits and vegetables are generally more expensive than staple carbohydrate foods like rice and they are not readily available on university campuses probably because they are perishable and not in high demand. Inadequate consumption of this group of foods may be because the students had limited funds to purchase them compared to the cheaper carbohydrate rich foods. About one quarter of the students stated inadequate funds as the major barrier to eating healthy and balanced meals. Limited resources have been highlighted in other studies to greatly compromise dietary patterns [17]. Inadequate fruits and vegetable intake have been singled out as a behavioral risk factor in the development of non-communicable diseases [20].

Almost 30 % of the respondents (29.0%) drank carbonated soft drinks daily while 25.0% drank it 4–6 times in a week. The prevalence of daily consumption of carbonated soft drink is similar to what was obtained in University of Ibadan, Nigeria, (27.1%) although lower than what was obtained in Cincinnati (51.2%) and North America (65.0%) [21, 22]. The major challenge with consumption of carbonated soft drink is the excessive consumption of refined sugar which can lead to obesity and hence other risk factors of NCDs. Research has shown that increase in prices of such drinks leads to a lower demand for them, increased demand for alternative drinks like non-sugary fruit juice and hence decrease in BMI and the prevalence of overweight and obesity [23]. Taxes and subsidies are likely to be an effective intervention to reduce consumption of carbonated soft drinks and other unhealthy foods associated with obesity and chronic disease, with evidence showing a consistent effect on consumption across a range of tax rates [24].

Almost half of the students ate pastry snack and ate it more than three times daily. This study is in congruence with others which showed that college students consume excessive calories from high-fat snack foods and fast food and eat little nutrient-dense foods (fruits, vegetables, and low-fat dairy) [25, 26]. Almost one quarter of the respondents ate in fast food restaurants daily indicating that at least seven [7] meals were taken away from home each week. This is similar to another study where most of the students ate average of 6.1 meals in restaurants every week [21]. Frequent visits to fast food restaurants are most likely because of their proximity to the students. Fast food restaurants have multiplied rapidly over the years due to nutrition transition and globalization; meanwhile, research has shown that proximity to fast food restaurant encourages its patronage [27, 28]. The challenge with pastry snacks and fast foods are high level of fat and calorie which are implicated as risk factors for NCDs.

Meat was more commonly consumed than fish. Only 30.0% of the respondents ate fish more than three times a week. Although this low proportion is higher than the proportion obtained from students in Azad University (47.2% ate fish once or twice a month), it is too low in view of WHO recommendation that people should eat fish at least three times weekly especially because of the healthy polyunsaturated fatty acids. There is an association between adequate fish intake and reduced risk of early and late age-related macular degeneration (AMD) [29].

In this study, the prevalence of abdominal obesity (by waist circumference or waist-to-hip ratio) was higher than general obesity. Meanwhile, abdominal obesity has been shown to be better predictor of NCDs than general obesity [30].

Family history of obesity or breakfast consumption were not associated with obesity in this study compared to other studies where family history of obesity and skipping of breakfast were predictive of overweight and obesity. Other studies consistently show that breakfast skippers are more likely to be obese than people who eat breakfast [31, 32]. However, alcohol consumption was positively associated with general obesity (based on body mass index). Alcohol consumption has been shown to be a risk factor for obesity and the association is a dose-response relationship [33, 34].

Low HDL-c was the most common dyslipidemia in the study population (57.3%) and was more frequent compared to another report obtained in South Western Nigeria (43.1%) and USA (20.1%) [35, 36]. The prevalence of high LDL-C (23.8%) was however consistent with reports from South Eastern Nigeria. (21.7%) [37]. Low HDL-C actually constitutes a higher risk factor for NCDs compared to high LDL-C [38]. The high prevalence of low HDL-C is most likely because most of the students consumed processed foods especially biscuits, snacks pastries, and margarine which have a lot of fat especially trans-fat more than whole and natural foods such as fruits, vegetables, and fermented foods which have pre-biotics and pro-biotics.

Moreover, many of them used vegetable or groundnut oil often which is most likely the commonly available cheap oil that are heat pressed and contains trans-fat. These snacks foods, margarine, and heat-pressed oil contain trans-fat which is implicated in reducing the healthful HDL-C and increasing the un-healthful LDL-C. Trans-fat also causes many other health problems such as obesity, metabolic disorders, and impairment of the metabolism of long-chain polyunsaturated fatty acids (LC-PUFAs). Excessive trans-fatty acid intake in pregnancy has been shown to reduce LCPUFAs levels in infants at birth, meanwhile LCPUFAs are responsible for the positive association between breastfeeding and intelligence [39, 40] On the other hand, prebiotics and probiotics which can be obtained from fruits, vegetables, and fermented foods have been known to lower cholesterol [41].

Raised LDL-C was associated with lower mothers’ level of education and worse methods of obtaining water which are proximate of the socio-economic status of the students. This means that the poorer students were more likely to have dyslipidemia. This is in consonant with recent finding which show that the earlier labeling of chronic diseases as “diseases of affluence” is increasingly a misnomer and there is a global shift of the burden of NCDs toward the poor [42].

The prevalence of hypertension in this study (2.8%) was similar to that of students in Central University in the West Bank (2.2%) but lower than obtained from another study in Makerere University where 54.0% and 11.0% had systolic pre-hypertension and hypertension and 43.0% and 18.0% were hypertensive [34, 43]. The prevalence of hypertension among these undergraduates was also lower than that obtained from other Nigerian studies {(20.8%) and (16.3%)} most likely because blood pressure increases with age and the other studies were conducted among adults [38, 40].

Abdominal obesity was statistically significantly associated with hypertension in this study even though high BMI was not associated with it. Abdominal obesity has been shown to be more highly correlated with the metabolic risk factors compared with elevated BMI. If obesity is defined in terms of waist-to-hip ratio (WHR) instead of BMI, the percentage of populations categorized as being at risk of heart attack will increase by threefold worldwide [44].

The prevalence of impaired fasting glucose (pre-diabetes) was 1%, but nobody had diabetes. This is very rare compared to other studies. For instance, a study among students of the University of Kansas showed that 9.0% had impaired fasting glucose while another study among adults in South Western Nigeria reported 2.5% [45, 46].

### Limitations of the study

Only chi-square test was employed in statistical analysis and this might not have been sensitive enough since many confounders could likely change the results of the association.

## Conclusions

Consumption of processed foods was high while that of fruits, vegetables, whole grains, and legumes was very low. This unhealthy dietary pattern along with alcohol consumption was associated with abdominal obesity. High prevalence of dyslipidemia was associated with low socio-economic status of parents. Undergraduate students are at risk of obesity and other metabolic risk factors of NCDs. Undergraduates in Lagos need to desist from alcohol consumption and consume more energy from whole foods that are rich in fiber for management of obesity. This result is useful for policy-makers in identifying specific areas where intervention is needed to improve diet and prevent metabolic risk factors of NCDs among university undergraduates, thus reducing morbidity and mortality in later adulthood and beyond.

## Abbreviations

HDL: High-density lipoprotein
LDL: Low-density lipoprotein
LMIC: Low-to-middle income countries
NCD: Non-communicable diseases
